# African American Couples’ Experiences during an Exercise Intervention Interrupted by the COVID-19 Pandemic: A Qualitative Case Study

**DOI:** 10.3390/ijerph19074190

**Published:** 2022-04-01

**Authors:** Lyndsey M. Hornbuckle, Wendy McLean Cooke, Amy Rauer, Cristina S. Barroso

**Affiliations:** 1 Department of Kinesiology, Recreation, & Sport Studies, University of Tennessee, Knoxville, TN 37996, USA; 2 Department of Child & Family Studies, University of Tennessee, Knoxville, TN 37996, USA; wmcleanc@vols.utk.edu (W.M.C.); arauer@utk.edu (A.R.); 3 College of Nursing, University of Tennessee, Knoxville, TN 37996, USA; cbarroso@utk.edu

**Keywords:** resistance training, walking, dyadic interviews, remote exercise training, videoconferencing

## Abstract

Exercise intervention researchers often struggle to transition participants from supervised/laboratory-based exercise to independent exercise. Research to inform this critical juncture remains underdeveloped. This qualitative case study investigated the transition from laboratory-based to home-based training in a subset of middle-aged and older African American couples whose exercise intervention experience was interrupted by the COVID-19 pandemic. All four couples (*N* = 8) whose study participation was interrupted participated in dyadic interviews by videoconference. Two investigators independently reviewed verbatim transcripts, and then used an iterative open coding approach to identify themes from the qualitative data. Three main themes were identified: (1) resistance training program modifications, (2) partner interactions, and (3) external pandemic-related factors. Each theme included both positive and negative feedback related to participants’ experiences. Overall, virtual, home-based training appeared acceptable and feasible in this group. Further research is needed to investigate the utility of virtual training to effectively transition participants from laboratory-based to independent exercise.

## 1. Introduction

As the world continues to cope with the COVID-19 pandemic and its enduring effects, researchers are also trying to adapt to procedural modifications forced by the related circumstances. Although the pandemic has caused unforeseeable interruptions to pre-set research protocols and interventions that had previously undergone meticulous planning [1], the current context provided investigators with an atypical opportunity to view their research projects and programs through a uniquely alternative lens. Study teams that are able to take advantage of this opportunity may also help mitigate some of the time and data losses that are unavoidable in the current research climate.

The potential research benefits of the opportunity presented by the pandemic were particularly relevant for exercise intervention researchers, who often struggle to facilitate a smooth transition for participants from the supervised, laboratory-based setting to independent exercise in order to foster an environment supportive of sustainable exercise behavior changes [2]. Research to inform this critical exchange point is vital, yet remains an underdeveloped area of the existing literature. The current investigators recently piloted an exercise intervention developed to increase exercise and improve cardiometabolic health in middle-aged and older African American (AA) couples [3] that was indeed interrupted by the COVID-19 pandemic. This population was studied because it is particularly vulnerable to various cardiometabolic health disparities [4]. It has been well established in the literature that cardiometabolic disease can be prevented and/or treated with regular physical activity [5,6]. Given concerns that the COVID-19 pandemic and its related social isolation have contributed to rising rates of physical inactivity in aging adults [7], identifying sustainable approaches to independent exercise for this population is both timely and critical.

Even pre-pandemic, facilitating a successful transition for aging participants as they progress from supervised training to independent exercise has proven challenging both in the current study team’s research and in other laboratories [2,8,9]. In the current authors’ research, post-study qualitative evaluations of the recent pilot exercise intervention uncovered participants’ perceptions of having received inadequate support when shifting to autonomous exercise in the study’s partner commercial gym at the completion of the supervised, laboratory-based portion of the program [8]. When the pandemic prompted an abrupt move from laboratory-based to in-home resistance training (RT) in a subset of pilot study participants, investigators deemed it important to assess those participants’ perceptions of the in-home experience. Given the distinction and the altered experiences of this group, a separate qualitative analysis of these particular participants’ experiences was warranted and important. Further, the feasibility and desirability of employing a virtual option to support participants’ progression to independent exercise behaviors in this population were unknown. Being able to obtain feedback about this transition would allow investigators to evaluate the value of a supervised, in-home training model as a potential transition tool that could be implemented more widely when participants leave the laboratory setting.

Therefore, the purpose of the current qualitative case study was to investigate the distinct experiences of transitioning from laboratory-based to home-based exercise training in the subset of AA couples whose intervention experience was interrupted by the COVID-19 pandemic. The current study was a part of a larger qualitative evaluation of these couples’ overall experience in the pilot study; however, the focus of this paper is to: (1) report these participants’ experiences unique to the circumstances while navigating the COVID-19 pandemic; and (2) discuss how investigators can use these data to inform future iterations of the pilot intervention, with specific focus on effectively transitioning participants from a research study to more autonomous exercise behavior.

## 2. Materials and Methods

### 2.1. Brief Overview of the Pilot Exercise Intervention (Pre-Pandemic)

The purpose of the pilot study was to examine a community-engaged, dyadic approach to increasing physical activity in middle-aged and older AA adults. The outcomes measured included exercise adherence and several cardiometabolic risk factors (e.g., body composition variables, blood pressure, and metabolic blood variables) with the overarching goal of developing of a sustainable exercise intervention that may help reduce cardiometabolic health disparities in the AA community. Extensive details related to the development, methods, and results of both the community-engaged pilot study [3,10] as well as the qualitative evaluation of participants who completed the pilot intervention before the pandemic [8] are published elsewhere.

To briefly summarize the pilot study methods, romantic male-female couples 40–80 years of age who were cohabiting were recruited. Both partners also had insufficient physical activity levels, defined by average daily step counts <8000 steps at baseline. All couples who entered the 12 week exercise intervention were randomly assigned to either exercise together (ET) with their partner or exercise separately (ES). A cluster block randomization strategy was used to ensure even distribution of the two treatments. The exercise prescription itself was identical for both groups and included purposeful walking for exercise (3×/week for ≥30 min/day) measured daily by both walking logs and a wrist-mounted activity monitor. Walking exercise was completed at the participants’ convenience using any chosen modality such as trails, neighborhood streets, or treadmills. In addition, all participants were asked to report to the University of Tennessee, Knoxville Exercise and Fitness Promotion Laboratory 2×/week (either together or separately, depending on group) to complete a progressive, circuit-style RT workout supervised by their designated exercise trainer. After a 5–10 min general warm up, the RT protocol was completed on 10 machines that included all major muscle groups. These exercises are included in Table 1. Participants were progressed from 2 sets (weeks 1–2) to 3 sets (weeks 3–12) of 8–12 repetitions for each exercise. The trainer led participants through 5–10 min of upper- and lower-body stretching at the conclusion of each RT session. The couples who exercised together also received education during their RT sessions on how to support their partner during exercise and enhance receptivity to partner support as part of their treatment. The couples who exercised separately did not receive educational intervention. Study outcomes included exercise adherence, the provision of partner support and receptivity to partner health influence, and multiple cardiometabolic risk factors.

### 2.2. Exercise Intervention Modifications Due to the Pandemic

It is well known that the COVID-19 pandemic prompted social distancing mandates and research restrictions worldwide. As the walking exercise prescription for the pilot study was originally designed to occur outside of the laboratory, no protocol changes were necessary except for a strong recommendation to comply with the Centers for Disease Control and Prevention guidelines for social distancing. In contrast, the RT protocol had to undergo extensive and immediate modifications, as a cohort of participants was 5 ½ weeks into the program when in-person research activities ceased at the University of Tennessee, Knoxville. Although it was impossible to design an identical program, the laboratory-based program was modified to create a safe, relatively comparable home-based total body program. Adjustable dumbbells were chosen to help carry out the modified protocol due to their user-friendly design that only required a dial turn to change the weight (i.e., no changing of weight plates). Further, this choice offered a wide range of resistance (5–52.5 pounds/dumbbell) which served partners with varied strength levels well and minimized required storage space in the home. 

Table 1 shows the home-based exercises chosen to be comparable to the laboratory-based exercises to which participants were physically accustomed. For safety, participants began the home-based program with body weight only or a modest amount of dumbbell weight until the trainer was satisfied that body positioning and lifting form were correct. The weight progression for each individual followed the same pattern as the laboratory program, where participants progressed from 2 sets to a maximum of 3 sets of 8–12 repetitions for each exercise in a circuit-style with ~2 min rest between sets. Each training session still began with a 5 min warm-up and concluded with light upper- and lower-body stretching. Exercise trainers monitored/coached sessions via Zoom© videoconferencing software (Zoom Video Communications, Inc.; San Jose, CA, USA). 

### 2.3. Participants

Purposeful sampling was used for the current case study. The current study’s “case” (participants whose study experience was interrupted by the COVID-19 pandemic) was chosen based on its timely relevance to the current research climate and its potential to inform future exercise interventions looking to provide transitional, post-study support. All pilot study participants whose participation was interrupted by the pandemic (four couples; *N* = 8) were invited to participate in dyadic interviews related to their overall assessment of the pilot exercise intervention as well as their transition from laboratory- to home-based RT. This interruption occurred at week 5 ½ of the 12 week program. All four of the eligible couples accepted the invitation to participate in the post-intervention qualitative interviews. 

All participants were emailed electronic copies of the informed consent form upon initial contact and informed that their verbal consent would be obtained prior to the interview if they chose to participate. Consent was given by all participants. All individual participants received an electronic $30 gift card from a national retailer for participation in the interview. All study procedures were reviewed and approved by the University of Tennessee, Knoxville Institutional Review Board.

### 2.4. Dyadic Interviews

Dyadic interviews were used in the current sample, which differed from the focus groups that were used pre-pandemic to conduct qualitative evaluations [8]. This decision was made as investigators were sensitive to the timing of the interviews (i.e., mid-pandemic in a highly-vulnerable, highly-affected demographic) and deemed it more appropriate to address participants privately by dyad versus using focus groups as had been done with the previous participants. The interviews were conducted remotely via Zoom© videoconferencing and were approximately one hour in duration. The principal investigator (LMH) greeted and reviewed the informed consent with all participants, after which they were asked to provide verbal consent. After consent was given by both participants in the dyad, the principal investigator exited the videoconference and a second research team member (WMC) conducted the interview. To encourage candid feedback/authenticity and improve trustworthiness of the study [11], the interviewer had not previously met the participants and had no previous involvement in the pilot intervention study. A brief demographic survey was then administered verbally. These data were combined with other selected data collected at the pilot study baseline to provide descriptors of the participants. 

The semi-structured interview guide used to evaluate the pre-pandemic pilot study couples was supplemented for use in the current study to include questions that would capture data related to participants’ study-related experiences while navigating the COVID-19 pandemic. Details related to the development of the original interview guide as well as the interview guide itself have been published [8]. Briefly, the interview guide was developed using a formative approach to process evaluation in order to collect data on participants’ lived experiences during the pilot intervention [12]. The interview guide contained questions that focused generally on the strengths, challenges, and cultural relevance of the pilot study. Secondary questions in most of these topic areas inquired about how the study could be improved in the future. Additional questions included in the interview guide for the current study focused on how different aspects of the study changed once the laboratory RT moved to the home setting with trainers providing guidance via Zoom©. Examples include: “*Please comment on any differences that you experienced when your training was shifted to remote communication through Zoom©.*” and “*Was training remotely using this platform [Zoom©] better, worse, or about the same as training in person? Why?*” For the majority of the interview, both individuals within each dyad were given the opportunity to respond to each question. Although answering individually, the nature of the dyadic setting allowed for and encouraged interaction between partners while each was recalling their perspective of the study experience. At the end of the interview, one partner was asked to leave the room and each individual was asked 2–3 questions (depending on intervention group) without their spouse present. This provided space for individuals to provide information about their experience without censoring due to the presence of their partner. 

Although a videoconferencing platform was used for communication ease and to provide a more sociable interaction, interviews were audio recorded only. All interviews were transcribed in real time using Otter.ai software (Otter.ai; Los Altos, CA, USA). Each digitally-produced transcript was then compared to its respective audio recording and manually corrected by two different research team members. The principal investigator then reviewed each transcript against the audio recordings and made any final edits for accuracy.

### 2.5. Data Analysis

The qualitative case study data derived from the interviews were analyzed using an open coding, thematic approach [13,14] by two investigators (LMH and WMC). A case study approach was used to describe the behavior and activities of the current participants and their motives given the unique circumstances surrounding the COVID-19 pandemic. The iterative coding process was collaborative in nature. An initial planning meeting was held to discuss case study as a research tool and to develop a research plan. An audit trail was used to reflect the team’s analytical memos, which aided in the process and study write up. Investigators then independently designated codes to the thoughts and views disclosed by participants. In the first round of collaborative data coding, team members were able to arrive at a consensus on the overall content of the transcripts. Next, team members coded each transcript individually, then held a second consensus meeting to compare codes and reach a consensus on the codes selected. In two subsequent meetings, the initial codes were reduced to categories and followed by themes to reflect participants’ overall perception of how they experienced the intervention after it was interrupted by the COVID-19 pandemic. The coding and reduction of data were facilitated by NVivo 1.0 software (QSR International Inc.; Burlington, MA, USA). 

## 3. Results

Table 2 shows descriptive characteristics of the participants at baseline of the pilot intervention. In general, participants were older (mean age 57.3 ± 10.4 years), categorized as obese (body mass index 33.6 ± 5.4 kg/m^2^), low active (5699 ± 1583 average steps per day) [15], and varied in terms of education and employment. Two couples participated per intervention group (ET: *n* = 4; ES: *n* = 4). All participants were married and cohabiting. 

Table 3 presents the three main themes that emerged from the qualitative data analyses, as well as categories and sub-categories within each theme. The themes were (1) resistance training program modifications, (2) partner interactions, and (3) external pandemic-related factors. Sample quotes supporting each theme are included initially within the text, and then more expansively in Table 3. 

### 3.1. Theme 1: Resistance Training Program Modifications

*“…at first, I was like, how are we going to do this on Zoom©? But it worked out.”*

—ES female (Couple #3)

Participants spoke about a variety of aspects related to their experiences with the unexpected and abrupt change from laboratory-based training on machines with a live exercise trainer to home-based training using dumbbells and body weight while interacting with their trainer by videoconference. The *Transition to Videoconference Training* category reflected both positive and negative experiences from the participants’ perspective. Both the videoconferencing itself and the platform chosen were apparently familiar and acceptable to most participants. This familiarity assisted with the ease with which the study team was able to transition participants to the new training format and program. That said, one couple chose to discontinue the RT portion of the study due to the change to videoconference interaction. Participants also commented on the differences in their interactions with their exercise trainers after moving the intervention online. Some participants reported no change in the quality of their interactions, whereas others expressed concern with the trainers’ ability to accurately assess their effort and evaluate proper movement form.

*“…with the [pre-existing] knee problem that I have…I don’t feel like I got the same um, workout…even with my knee problems, the machines, you could utilize those to help strengthen them. At home, I have…one of those big balls that you can sit on and I would use it to support my back and roll up and down the [wall], but it just wasn’t the same.”*

—ET female (Couple #4)

*New Resistance Training Modality* was the second category within this theme cited to have affected the participants’ experience. In general, participants expressed a preference for the machines used in the laboratory when compared to the adjustable dumbbells used after the transition to home-based training. One ET couple also identified issues related to the time required to change the dumbbell weight values when alternating sets between partners.

### 3.2. Theme 2: Partner Interactions

*“I’m pretty sure if y’all actually have the old Zoom© videos of her working out you’ll see me in the background cheering her on. Because I am her…I am her biggest fan you know. So whenever she does anything I’m right there to cheer her on, you know.”*

—ES male (Couple #3)

Participants also reported both positive and negative aspects of their interactions with their partners after the transition to home-based training. These varied perspectives were presented in the *Perpetuated Positive Interactions* and *Shift in Couple Unification* categories. Although couples in both the ET and ES study groups reported that positive interactions related to study participation (e.g., encouragement, pride) continued and even flourished in the home-based training program during the pandemic, it was apparent that some partners experienced a shift in their ability to participate in the workouts as a unified couple.

### 3.3. Theme 3: External Pandemic-Related Factors

*“Well, I think the problem I faced was the fact that our routines were so much different from what they might normally be because we were participating [in the study] during all this other drama that’s going on in the world. So, it changed—changed who we were as participants* *.”*

—ET male (Couple #1)

Given the fundamental and numerous challenges that agitated nearly every aspect of day-to-day life at the onset of the COVID-19 pandemic, it was not surprising that participants cited factors outside of the study team’s control or not directly related to the study that affected their study experience. The category *Waiting to Transition from Laboratory to Home-Based Resistance Training* presented two critical issues that presented challenges for participants when they were able to resume exercise. For context, there was an approximately three-week waiting period between the mandated cessation of in-person data collection and the relaunch of the remote RT program. This period was necessary for the study team to re-design and acquire Institutional Review Board approval for the at-home RT program, re-train the exercise trainers, communicate the new program with participants, and purchase and distribute new equipment to participants. Participants reported a loss of fitness gains and a loss of routine during this period, which was inherently disruptive to the flow and cumulative gains of the program. *Unforeseeable Circumstances* connected with pandemic-related changes in the personal lives of participants were also an apparent stressor to the study experience. The circumstances surrounding the pandemic simply changed how participants were able to engage in the study. Additionally, given the undesired weight gain that has been reported since the start of the pandemic [16,17], *Pandemic Weight Control* was cited as a perk of study participation.

## 4. Discussion

The current study provides a unique supplement to the comprehensive pilot work referenced as it examines a distinctive subset of participants whose exercise intervention program was abruptly interrupted by the COVID-19 pandemic. When this occurred, investigators were forced to immediately decide whether to adapt the intervention for remote implementation, or to discontinue RT altogether. The current research team found merit to pivoting the intervention and wanted to continue serving the participants who were reluctant to discontinue the study. 

Our decision was also informed by a recent review of physical activity interventions in AA women that spanned 15 years [18]. This review suggested that home-based activity programs were a promising method to promote physical activity as this approach removed some of the barriers associated with physical activity adoption. Another review of physical activity interventions in marital dyads showed that each of the randomized trials reviewed (*N* = 6) were delivered in person [19]. This exhibited a lack of available data examining other methods of couples-based exercise intervention delivery. One last study that examined the effects of an exercise intervention on adherence in older Japanese adults showed no significant long-term adherence for the home-based RT program that followed an 8 week supervised exercise program in either intervention group (one group of married couples exercised together and one group of individuals exercised alone) [9]. As such, investigators believed implementing and qualitatively evaluating a home-based program could benefit the overall knowledge base in this area. This paper provides valuable information related to middle-aged and older AA participants’ perceptions of the intervention study experience while exercising at home. These data can inform future exercise research related to the transition of participants from a laboratory-based research study to a more autonomous exercise routine.

### 4.1. Interpretation of Themes

The data that support Theme 1 collectively conveyed participants’ mixed feelings about transitioning to the home program. Interacting with the exercise trainer via videoconference was well received overall and surprisingly required no additional training for any of the couples. Multiple participants noted comparable interactions with their trainer through videoconference, which was encouraging for the possibility of future use in home-based protocols. In contrast, one couple’s desire to discontinue home-based RT was based upon having to participate using videoconferencing and their concerns with it being feasible in their home. Additionally, the equipment change from machines to dumbbells was not favorable for many participants for various reasons largely related to unfamiliarity. This aligns with previous data in older adults showing a higher preference for machine-based RT compared to free weights [20]. Although there are clear health and functional benefits with any type of muscular overload [21], machines can provide a relatively safe and comfortable method for inexperienced and/or deconditioned individuals to adopt RT as they help control posture and body position. Machines can also be adjusted to heavier weight loads quickly with minimal effort. 

Investigators speculate that these participants’ pre-study inexperience with exercise in general, as well as their low physical activity levels and the myriad of external stressors related to the pandemic, may have had a negative impact on participants’ perspectives related to the transition in general. Again, this is understandable given couples had originally agreed to and expected a machine-based laboratory program with live support from a trainer, versus a home-based program using body weight and dumbbells supervised through videoconference. In addition, the abrupt nature of the change may have invoked some negative feelings that could have been mitigated had the transition to home-based exercise been planned and participants been trained for the change in equipment. Findings suggest that investigators looking to use remote strategies for exercise training should adequately prepare participants both mentally and physically (e.g., acclimate to the specific exercises and equipment) to transition to a remote program. The willingness of participants to shift their training modality is promising as it suggests that even in a non-ideal situation, middle-age and older AA adults may be open to participating in a remote exercise program. Further research should examine whether the current study’s acceptability is consistent in a larger sample and in aging couples of different demographics. Researchers might also examine the acceptable length of time for this method of intervention delivery and whether particular personality types and/or couple dynamics foster positive or negative experiences and functioning in the remote training environment.

Considering the effects of couple dynamics seems particularly important given that Theme 2 highlighted various interactions that occurred within each couple as a result of the shift to home-based RT. Investigators note that the stress of the circumstances surrounding the pandemic appeared to perpetuate the positive tone or exacerbate the negative tone of the couples’ existing relationship. Such a finding is consistent with recent theoretical and empirical work suggesting that the pandemic has amplified many of the previous stressors and relationship dynamics for couples [22]. Those who were doing well prior to the pandemic were able to maintain their relationship satisfaction, whereas couples whose relationships were less harmonious before experienced exacerbated conflict [23]. As it pertains to the current study, the onset of the pandemic either prompted the couples to continue working together while participating in the study, or facilitated a gradual unraveling in terms of their perceptions of the study experience. 

Interestingly, group assignment did not present as a factor in the perception of the study experience in this sample. Instead, it appeared that different aspects of the transition dominated for each couple and shaped their experience and attitude toward the program. For example, the conflict and study dissatisfaction that was newly introduced in the home setting for one couple appeared to stem largely from the change in RT equipment and modality. This may suggest that they valued the laboratory equipment and workouts. That said, external factors secondary to the delays and stress of the pandemic also presented as underlying issues for this couple. This finding is concerning as external factors hampered the program benefits and reduced exercise enjoyment, both shown to be barriers to behavior change [24]. As mentioned earlier, the unique circumstances that prevented adequate preparation for the transition likely played a role and can be corrected in future transitions. Future research should also examine whether virtual training is conducive to exercising together with a partner.

Theme 3 presented several pandemic-related factors that affected participants’ perception of the study experience. The loss in exercise time while study staff worked to prepare to transition participants to the home-based program was unavoidable, yet disappointing for both investigators and participants. The following inevitable loss of fitness gains and the exercise routine that had been developed over the initial 5 ½ weeks of the program was certainly disruptive for all participants, yet they expressed varying levels of frustration. For a few, this was communicated in tandem with annoyances in acclimating to the new exercises and equipment. Despite education and encouragement meant to soften self-expectations while adopting the new home-based program, some participants attributed the initial physical assimilation period to the weeks spent not training. Again, this issue will be mitigated with transitional training in future programs. Consistent with most individuals worldwide, participants also cited life, routine, and mindset changes as factors affecting their study experience at the onset of the pandemic. Specific circumstances varied widely both within and across couples, but can be summarized as new and unforeseeable responsibilities and broad life modifications. Finally, it is encouraging to learn that weight control during this challenging and inherently more sedentary time was a positive impact of study participation.

Life course theory can help guide the interpretation of these qualitative data [25], as it can illuminate the effects of transitions on couples [26,27]. Life course theory recognizes that individual lives are influenced by their historical context. As such, this framework can be used to guide health-related interventions with a focus on fostering an environment that supports healthy behaviors. In the current study, life course theory was used to help investigators interpret how a major stressor (the pandemic) affected the transition of an exercise program.

Two key life course theory concepts illustrated in the current study included environment and timing [28]. The idea that the general community environment, inclusive of the social environment, has an effect on one’s ability to be healthy is highly relevant in the context of a worldwide pandemic. Not only is there the inherent risk of acquiring an infectious disease, but the subsequent social distancing mandates and stay-at-home orders themselves had negative effects on the physical and mental health of those adhering to these mandates. As it relates to the current study, the disruption in daily routines and restrictions in exercise venue access limited the choices related to how participants could safely obtain regular exercise. It is recognized that individuals are still struggling with these challenges as the pandemic persists. The current data also reflect the life course concept that health behaviors can be particularly vulnerable during susceptible periods of time. This is apparently relevant in the current climate for reasons cited above.

### 4.2. Limitations

The investigators acknowledge some limitations of this study. Although the home-based exercises were chosen in an effort to provide comparable dynamic exercises using muscle groups similar to those utilized on the laboratory machines, participants were unaccustomed to the at-home program that utilized body weight and dumbbells, which required/engaged core stability and balance [29]. Even after employing the appropriate progressions and having 5 ½ weeks of RT established, there were inevitably fitness losses in the 2 ½ week transition period where the study team was preparing to launch the home-based program. As such, participants unfortunately had to adapt to a new modality with no recent training with body weight/dumbbell loaded exercises. This, plus the fact that the original and post-interruption modalities were different, compromised training continuity and disturbed various aspects of the program. 

Although this case study included the study’s entire sample of participants whose exercise intervention was interrupted by the COVID-19 pandemic, only four couples experienced this disruption and thus were interviewed. Even so, this study provides novel qualitative data about an unprecedented circumstance that may help exercise interventionists moving forward. Additionally, the use of dyadic interviews compared to one-on-one could have prevented participants from fully disclosing certain feelings about their study experience, particularly as it related to their partner [30]. To minimize this possibility, investigators provided the opportunity for all participants to provide feedback individually without their partner present. Investigators employed various methods to avoid the risk of analytical biases including the use of an interviewer previously unknown to the participants and not involved in the study development. Additionally, several consensus meetings attended by two different investigators (one who was present for the interviews and one who was not) were conducted, which allowed for reflexivity between the investigators to confirm the findings and increase the trustworthiness of the study [11,31]. Last, it is recognized that these data are not generalizable to all middle-aged and older adults or AA adults. That said, the data may be relevant and informative in these and comparable populations.

## 5. Conclusions

### Overall Assessment and Future Direction

In summary, these data as well as the pre-pandemic qualitative data linked to the same pilot study [8] indicated that middle-aged and older AA participants felt some level of vulnerability when the laboratory setting was removed. This suggests that planned transitions should include a psychoeducational component aimed at addressing this vulnerability. Although the context of the two data sets/groups differed (i.e., a planned transition to a commercial gym versus an abrupt exercise interruption then shift to home-based training), this key similarity will be addressed in the current laboratory moving forward and will hopefully inform other laboratories working with previously inactive aging adults. The current investigators learned key lessons that will likely be important for future exercise intervention research in this population, and offer examples of how these lessons can be employed. These lessons are as follows:

1. Utilizing a virtual training format appears largely acceptable and feasible in this group. However, participants should be adequately prepared for this method of program delivery (e.g., confirm or ensure comfort and home feasibility for videoconferencing, hold “practice” virtual training sessions to troubleshoot any barriers).
2. Participants must be trained for any changes to programmatic exercises and equipment. Although this is a well-established, foundational concept in exercise laboratories, the atypical circumstance of the pandemic forced hurried protocol changes with only virtual participant contact. While we hope not to facilitate a transition like the one experienced in the current study again, these data highlighted the need for enhanced support, grace, and encouragement when asking aging participants to commence any program with more independence.

Ultimately, the collective lessons learned from participants’ qualitative feedback were highly beneficial and highlight the need for prior planning and preparation when attempting to provide transitional support for participants as they complete any supervised exercise program. This information is relevant for future plans to support a more seamless transition for participants from a laboratory or other supervised setting to sustainable independent exercise in the current investigators’ research, even when the pandemic is no longer a concern. The current investigators also hope these data will stimulate the interest of other laboratories in order to advance science in this particular area of exercise intervention and adherence research. More research is needed to investigate the utility of virtual training to facilitate the transition from laboratory-based to independent exercise behavior, particularly for aging individuals who may be more comfortable participating at home. 

## Figures and Tables

**Table 1:** Comparison of resistance training program before and during the COVID-19 pandemic.

^#^ Laboratory-Based Machine Exercises (Pre-Pandemic)	* Home-Based Exercises with Dumbbells (During the Pandemic)
Leg press	Squats
Leg extension	Standing (or walking) alternating lunges
Leg curl	Romanian deadlifts
Chest press	Supine chest press
Shoulder press	Seated shoulder press
Lat pulldown	Standing row
Biceps curl	Seated biceps curl
Triceps press	Seated overhead triceps extension
Abdominal crunch	Supine abdominal crunches or partial curl-up from seated
Back extension	Prone back extension

^#^ All laboratory-based machine exercises were performed in a seated position. * Modifications were used as individually needed and appropriate during home-based program (e.g., assisted movements, body weight only, etc.).

**Table 2:** Descriptive characteristics of participants at intervention baseline (*N* = 8).

Variables	Mean ± SD or *n*
**Age (years)**	57.3 ± 10.4
	(range 41–72)
**Time in Current Relationship (years)**	28.5 ± 4.7
	(range 23–36)
**Body Mass Index (kg/m^2^)**	33.6 ± 5.4
	(range 25.7–42.7)
**Physical Activity (baseline steps/day)**	5699 ± 1583
	(range 3216–7843)
**Self-Identified Gender (*n*)**	Female: 4
	Male: 4
**Highest Education Level (*n*)**	Some high school: 1
	High school graduate or GED: 0
	Some college: 3
	Associate’s degree: 2
	Bachelor’s degree: 1
	Master’s degree: 1
**Employment Status (*n*)**	Employed full time: 3
	Employed part time: 1
	Self-employed: 1
	Out of work <1 year: 1
	Retired: 2
**Gross Annual Household Income (*n*)**	$75,000–$99,999: 2
	$100,000 or more: 4
	Preferred not to answer: 2
**Relationship/Household Status (*n*)**	Married, with dependents: 6
	Married, no dependents: 2

Data represent two couples in each pilot exercise intervention group (i.e., exercised together [*n* = 4] and exercised separately [*n* = 4]).

**Table 3:** Identified themes, categories, and sub-categories related to the interruption of the exercise intervention (*N* = 8).

**Theme 1: Resistance Training Program Modifications**	
Transition to videoconference training	* SAMPLE QUOTES
Initial reactions	“I was already familiar with Zoom© anyway. And…so once we got online [began training remotely] t’was just making sure I had the [computer tablet] in a place that picked up the biggest viewing ability for her [the exercise trainer].”—ET female (Couple #4)
	
	“You know, it’s very convenient to not have to leave the house. To be able to be trained online. That was great!”—ET male (Couple #1)
	
	“…even though we have a basement with workout equipment, based on the setup and the convenience and the comfort of training downstairs, it just wasn’t a good fit for us.”—ES female (Couple #2)
	
Interaction with exercise trainer	“Well, I think she [exercise trainer] did an excellent job when she moved us online. We had no problems contacting her. We had no problems, you know, getting input from her when we were exercising and all that. Ya know, plus encouragement. I, I think she did a wonderful job.”—ET male (Couple #4)
	
	“…the same way he [exercise trainer] pushed me when we were in person, he actually pushed me like that on Zoom©.”—ES male (Couple #3)
	
	“…when we were on Zoom©, to me it was hard for her to actually see. Even though she can see…you don’t get as much…so they can see how you’re doing. Whether you’re doing it [exercise movements] right or wrong.”—ET female (Couple #4)
	
	“…she [exercise trainer] didn’t have any way of knowing up close and personal, what, what effort was being required to do what we were asked to do.”—ET male (Couple #1)
	
New resistance training modality	
Experience with adjustable dumbbells vs. machines	“And sometimes I struggle with the weights. But um…I was used to ‘em [the laboratory resistance training machines]. But when you’re doing weights at home it’s kind of different it seems like. Um, but other than that, I mean, it was still a good workout.”—ES female (Couple #3)
	
	“I think you can gauge your progress so much more effectively with the apparatus [laboratory resistance training machines]…Like, yeah, it was like, it just wasn’t the same. It was a totally different experience.”—ET male (Couple #1)
	
	“…with the new weights you are using your own body as a hydraulic which is…it is more challenging.”—ET female (Couple #1)
	
Increased wait time between sets	“So, we’d have to take the time to adjust the weights then change it out, then change it back after she [partner] did her reps. And so, it was just more tedious.”—ET male (Couple #1)
**Theme 2: Partner Interactions**	
Perpetuated positive interactions	* SAMPLE QUOTES
Sustained encouragement to continue exercise	“Once we left the gym and came here to the house, like she said…as soon as we got the equipment that we needed to continue on. I mean it’s like you’re already doing this, you might as well keep on doing it.”—ET male (Couple #4)
	
Expression of pride	“Based on how she’s responded to the study…I’m really proud of her, that she’s been able to stick to the walking [during the pandemic]…”—ES male (Couple #2)
	
Shift in couple unification	“Well, we had that commonality [prior to the interruption]. We were on the same page. We looked forward to it [exercising together in the laboratory]….I just don’t know what happened. It [exercising together at home] became a, ‘You’re going too fast. You’re not doing this right.’…There was a lot of friction.”—ET female (Couple #1)
**Theme 3: External Pandemic-Related Factors**	
Waiting to transition from laboratory to home-based resistance training	* SAMPLE QUOTES
Loss of fitness gains	“I feel like all the benefits of what we work[ed] for, for the first six weeks, were lost.”—ET male (Couple #1)
	
Loss of exercise routine	“So I think the challenges were, you know, we had to wait to get the equipment. So trying to make sure that we kept our own selves accountable…uh, until we could get some kind of assistance in our home.”—ET female (Couple #4)
	
Unforeseeable circumstances	“…everything just changed because the whole situation in our life changed. My mindset changed. The fact that my [previously ill] parents [became inaccessible] and a lot of stuff happened. So uh, we just got in that fog like most people did during COVID.“—ET female (Couple #1)
	
Pandemic weight control	“…I will say, because a lot of people have complained about the COVID-19 pounds that they gained, I have gained weight but I can’t imagine what I would have gained had I not continued to do the walking and getting some form of exercise.”—ES female (Couple #2)

ET = exercised together group; ES = exercise separately group. * Quotes not included in the main text.

## Data Availability

Data are available to the publisher upon request for any verification purposes. Any data sharing must first be approved by the University of Tennessee, Knoxville Institutional Review Board. Data are not available to the public.
